# The role of fetal-maternal microchimerism as a natural-born healer in integrity improvement of maternal damaged kidney

**DOI:** 10.1590/S1677-5538.IBJU.2017.0324

**Published:** 2018

**Authors:** Abdol-Mohammad Kajbafzadeh, Shabnam Sabetkish, Nastaran Sabetkish

**Affiliations:** 1 Pediatric Urology and Regenerative Medicine Research Center, Section of Tissue Engineering and Stem Cells Therapy, Children's Hospital Medical Center, Tehran University of Medical Sciences, Tehran, Iran

**Keywords:** Fetal Stem Cells, Y Chromosome, Technetium Tc 99m Dimercaptosuccinic Acid, Green Fluorescent Proteins

## Abstract

**Purpose:**

To identify the fetal stem cell (FSC) response to maternal renal injury with emphasis on renal integrity improvement and Y chromosome detection in damaged maternal kidney.

**Materials and Methods:**

Eight non-green fluorescent protein (GFP) transgenic Sprague-Dawley rats were mated with GFP-positive transgenic male rats. Renal damage was induced on the right kidney at gestational day 11. The same procedure was performed in eight non-pregnant rats as control group. Three months after delivery, right ne- phrectomy was performed in order to evaluate the injured kidney. The fresh perfused kidneys were stained with anti-GFP antibody. Polymerase chain reaction (PCR) assay was also performed for the Y chromosome detection. Cell culture was performed to detect the GFP-positive cells. Technetium-99m-DMSA renal scan and single-photon emission computed tomography (SPECT) were performed after renal damage induction and 3 months later to evaluate the improvement of renal integrity.

**Results:**

The presence of FSCs was confirmed by immune histochemical staining as well as immunofluorescent imaging of the damaged part. Gradient PCR of female rat purified DNA demonstrated the presence of Y-chromosome in the damaged maternal kidney. Moreover, the culture of kidney cells showed GPF- positive cells by immuno- fluorescence microscopy. The acute renal scar was repaired and the integrity of dam- aged kidney reached to near normal levels in experimental group as shown in DMSA scan. However, no significant improvement was observed in control group.

**Conclusion:**

FSC seems to be the main mechanism in repairing of the maternal renal injury during pregnancy as indicated by Y chromosome and GFP-positive cells in the sub-cultured medium.

## INTRODUCTION

Fetal maternal cell trafficking (FMCT) can be defined as the presence of cells originating from genetically distinct individual without evidence of immunological response. FMCT is considered to be the trafficking of semi-allogenic fetal cells into the maternal circulation that may culminate in a mixtue of both maternal and fetal cells in maternal tissue during and after pregnancy. Several studies have demonstrated the persistence of FMCT in the CD34+ population for more than 30 years after delivery (1). Male cell markers have been applied in most studies because of its simplicity in identification of FMCT. In addition, FMCT is derived from both male and female fetus (2). Immune tolerance of the mother to the fetus and vice versa also appears to develop by this phenomenon (3). Migration, engraftment and differentiation of fetal stem cells (FSCs) to several maternal tissues during the pregnancy may happen, especially in damaged host organ (4–6). FSCs may have a crucial role in healing maternal damaged organs during the pregnancy by passing through the placenta and entering the maternal blood circulation. FSCs have also the ability to migrate to sites of the affected maternal organs, differentiate, and proliferate locally.

However, methods to determine the role of fetal progenitor cells in treating damaged organs during the pregnancy are still laborious. The accretion of FSCs in the local damaged organs may be due to the consequence of the disease; or caused by the response to tissue injury. Furthermore, it has been considered that FSCs have the ability to gain tissue specific markers as they migrate to the environment of damaged maternal organ (7,4). To date, the role of FSCs in functional improvement of the damaged organ has not been well evaluated.

In the current animal model, we used transgenic male rats expressing green fluorescent protein (GFP) in order to achieve the best sensitivity for better detection of FSCs in the maternal damaged kidney. The aim of the current study was to investigate the multilineage capacity of FSCs in repairing of maternal injured kidney as well as improving its functional capacity in a rat model of FMCT by detecting Y-chromosome and GFP-positive cells in the damaged part of maternal kidney.

## MATERIALS AND METHODS

### Animal model of FMCT and the subsequent induction of renal damage

The local ethics committee approved the experimental protocol. The principles of laboratory animal care (NIH publication no. 85-23, revised 1985) were respected for animal treatment.

Eight non-GFP female Sprague-Dawley rats weighting 220-310g were mated with GFP-positive male rats. All rats were maintained in standard single cages on a 12h darkness/12h light cycle with the best access to standard feed and water ad libitum in our laboratory. The rats were examined at 8h interval for detection of vaginal plague. The identification of vaginal plague was considered as gestational day 0 (GD0). Afterwards, female rats were kept in separate cages with consummative diet.

Renal mass ablation by specially designed diathermy or electrocautery probe has been employed by several studies (8–11). At GD 11, the rats were anesthetized by administering intraperitoneal Ketamine (40mg/kg) and Xylazine (4mg/kg) and the right lateral flank was shaved and swabbed with a solution of 0.5% chlorhexidine in 70% alcohol (Hibitane^®^). For right kidney exposure, a lateral incision was made before separating the kidney from the adrenal gland and perirenal fat. Then, the renal damage (focal burning) was performed on the lateral upper pole of the right kidney. The degree of burning could be precisely controlled by the period of contact with the kidney. In summary, the right upper renal pole was cauterized for a period of 4 seconds by the application of conventional cautery to avoid the incidence of necrosis as an irreversible renal injury. The maintenance of anesthesia was obtained by the injection of 0.75mg/kg Ketamine with 20 minutes intervals. The rats were then kept in specific cages under intensive care for termina-ting the gestational period. The same procedure was also performed in eight non-pregnant rats as control group to compare the role of FSCs in repair of damaged maternal kidney.

### Immunohistochemistry and immunofluorescence of fixed frozen samples

After 3 months of delivery, the rats underwent a nephrectomy surgery by the same surgeon in order to dissect the injured kidney. The kidneys were perfused with phosphate buffered saline to remove the red blood cells and were stained with anti-GFP antibody for identification of the FSCs in the non-GFP maternal damaged kidney. Anti-GFP antibody was purchased from Dako^®^ (Trappes, France). In summary, deparaffinized and rehydrated samples underwent antigen retrieval by the application of Tris/EDTA buffer (pH=9.0) and a vegetable steamer. Endogenous peroxidase was blocked with horseradish peroxidase (HRP). In the next step, slides were blocked in 10% normal serum with 1% bovine serum albumin (BSA) in TBS for 2h, after being washed in Tris buffered saline (TBS) plus 0.025% Triton X-100. Ap-propriate dilution of antibodies was applied for overnight incubation of the slides.

The fresh frozen samples were also analyzed by immunofluorescent microscopy to verify the cell trafficking of GFP-positive cells in the kidney of non-GFP rat. Other organs, including liver and lung were also analyzed by immunofluorescent microscopy in order to detect homing of GFP-positive cells in undamaged tissues.

### PCR

For PCR of the Y chromosome which was performed on genomic DNA, upper pole renal DNA was extracted using the QIAamp^®^ DNA kit (Qiagen AG, Basel, Switzerland) after nephrectomy of the damaged kidneys. The ubiquitous β-globin gene was amplified by PCR for the purpose of controlling the quality of extracted DNA and the lack of PCR inhibitors (12). Y chromosome DNA amplification was performed by the application of 1 microgram of extracted DNA by means of a single PCR assay with the primer set CNX43-F (5'-TTC CTT TGA CTT CAG CCT CC-3'), CNX43-R (5'-GTG TTA CAG CGA AAG GCA G-3'), KH-1F (5'-GAG AGA GGC ACA AGT TGG C-3'), and KH-1R (5'-GCC TCC TGG AAA AAG GGC C-3'). The PCR steps included denaturation at 94°C for 4 minutes, 38 cycles of amplification, and elongation at 72°C for 10 minutes. After electrophoresis with a 1.5% agarose gel at different temperatures, ethidium bromide staining was utilized to observe the results under UV trans-illumination.

### Cell Culture

After dissection of the damaged kidney in sterile condition, the upper pole of the kidney was cut into 5- to 10mm-thick coronal slices. The fragments were washed in chilled basal medium. The fragments from the damaged section were minced into 1- to 2mm pieces with crossed blades. Tissue fragments (an approximate amount of 5mL) were transferred to a tube containing 20mL of warm collagenase-trypsin solution. The tissue was incubated in an orbital shaker with gentle agitation within an incubator at 37°C for 1h. Subsequently, 20mL of basal medium contai-ning 0.05mg/mL DNase was added and the supernatant was gathered at 20-min intervals with gentle trituration. The collected supernatant was diluted with an equivalent volume of complete culture medium, dispensed into aliquots in 50mL tubes, and centrifuged at 100g for 15 min. Then, each pellet was resuspend in 45mL of complete medium and seeded 15 into one 75cm^2^ flask. The sub-cultured cells were analyzed by immunofluorescence microscopy in order to detect the GFP-positive FSCs.

### Technetium-99m DMSA renal scan *and single-photon emission computed tomography (SPECT)*

After induction of renal damage and three months after delivery in experimental group and three months after renal damage in control group, Technetium-99m-DMSA solution was injected into the tail vein of rats to determine the differential renal function of the damaged kidney in four rats of each control and experimental groups. The mean renal function was obtained in both groups. As DMSA solution needs approximately 4-6 hours to travel around the blood stream to reach the kidneys, anesthesia was maintained during this period. The progress of the DMSA solution through the kidneys was traced with a single-head rotating camera. For SPECT, an Elscint^®^ SP-1 computer was applied to produce images representing slices through the kidneys in different planes in four rats of each control and experimental groups. The images obtained by SPECT are functional in nature rather than being purely anatomical.

## RESULTS

**Figure 1:**
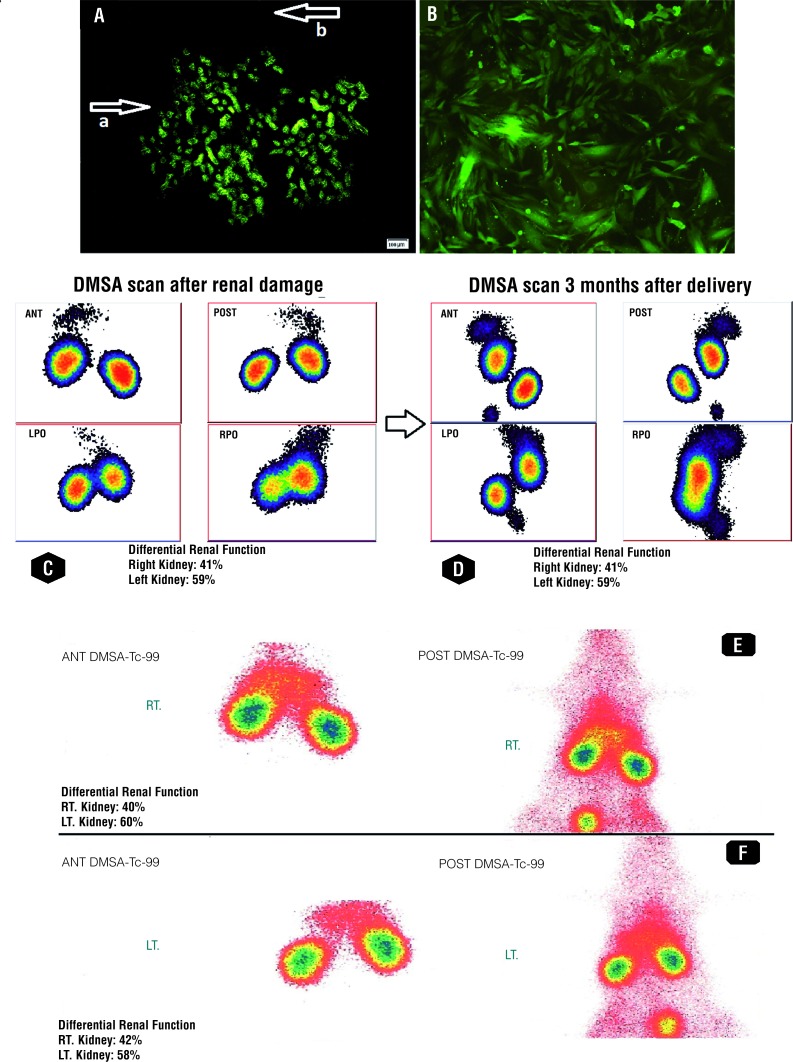
(A) Immunofluorescence of damaged part of maternal non-GFP kidney. Arrow a shows the damaged part of maternal kidney which is covered by GFP-positive FSCS. Arrow b shows the undamaged non-GFP maternal kidney; (B) Fetal GFP-positive cells in company with maternal non-GFP renal cells after culture of the damaged part from the upper pole. Technetium-99m-DMSA scan of pregnant rats (experimental group); (C) differential renal function after induction of renal damage confirmed decreased renal function on right kidney; (D) Renal function was similar in both kidneys three months postoperatively. Technetium-99m-DMSA scan of non-pregnant rats (control group); (E) differential renal function after induction of renal damage confirmed decreased renal function on right kidney; (F) Renal function did not improve significantly three months after renal damage induction

All rats survived the whole period of our study. The presence of FMCT in renal papilla and tubular epithelial cells was confirmed by immu-nohistochemistry staining and immunofluores-cence analysis of the damaged kidneys. These results indicated that fetal cells were present in the maternal peripheral circulation and they also contributed to the repairing process of maternal kidney by migrating to the damaged parts for further differentiation. As depicted in Figure-1 (A) GFP positive cells formed both tubular and interstitial lesions. However, no detectable focal aggregation of GFP-positive cells was found in immunofluorescent microscopy of lung and liver as samples of undamaged organs.

The presence of Y-chromosome in the damaged maternal kidney was obviously proved by the gradient PCR of female rat purified DNA. Regarding the fact that the exact temperature for PCR of this specific primer was unknown, gradient PCR method was applied for a purified DNA sample at different temperatures. Results of the gradient PCR showed that the most appropriate temperature for this specimen was 54°C. Gradient PCR at 49°C was applied in order to reassure the specific and non-specific binding of the primer.

The sub-cultured cells from the damaged part of the non-GFP kidney were viewed with immunofluorescent microscopy. The GFP-positive renal cells were observed in company with undamaged maternal renal cells (Figure-1B).

**Figure 2:**
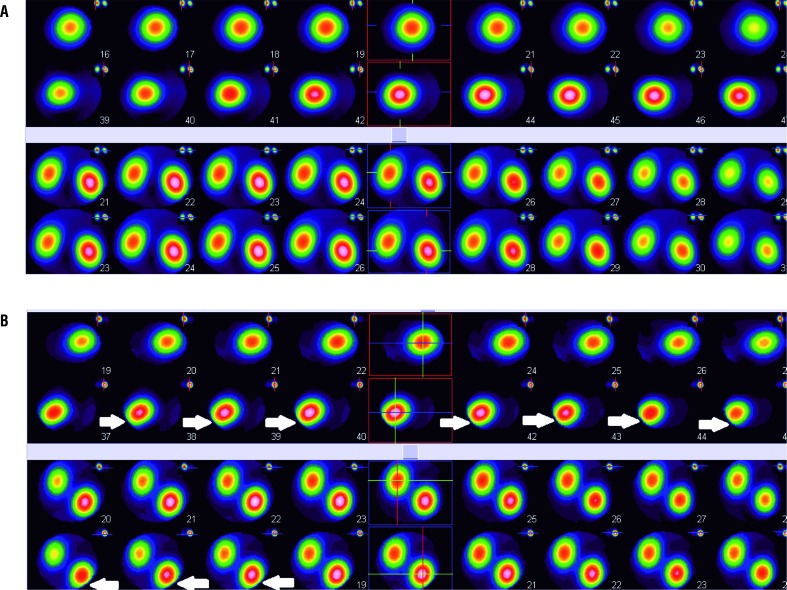
(A) SPECT in the experimental group three months after delivery showed satisfactory results without any significant impairment as compared with control group; (B) SPECT in the control group showed that the renal injury persisted after three months of damage induction. The arrows demonstrate the damage in several sections while such impairment was not detected in experimental group.

Renal imaging was performed by the application of Technetium-99m-DMSA solution the high percentage of which in the renal cortex results in the high gamma flow. Each of the static scintiphotos was obtained by the application of a high-resolution collimator 1 hour after administration of Technetium-99m-DMSA solution. The result of DMSA scan after induction of renal damage in experimental group revealed decreased renal function in the right kidney in which the damage was induced (41%±0.9% versus 59%±0.8%). However, similar uptake in both damaged (right) and normal kidneys (left) with uniform distribution pattern of renal activity was obtained 3 months after delivery (49%±0.7% versus 51%±0.4%). The results of DMSA scan in control group revealed no signi-ficant improvement in function of right kidney (mean±SD) after 3 months of renal damage in-duction (40%±0.3% versus 60%±0.7% after renal damage and 42%±0.8% versus 58%±0.6%, 3 months postoperatively). DMSA scan of pregnant and non-pregnant rats confirmed the role of FSCs in repairing the damaged part of the kidney (Table-1). The split renal function described in the DMSA scan was from average data of the groups (Figures 1-C-F). The results of DMSA scan were compatible with the outcomes obtained from SPECT. SPECT of control group showed renal damage in several continuous sections while satisfactory results without any detectable renal impairment were obtained in none of the sections in experimental group 3 months after operation (Figure-2).

**Table 1:** Comparing the renal function after renal damage induction and 3 months postoperatively in pregnant (experimental group) and non-pregnant (control group) rats.

		Experimental group		Control group		p value
		Right kidney	Left kidney	Right kidney	Left kidney	
Mean ± SD DMSA Renal scan (%)	After renal damage induction 3 months after renal damage	41±0.9	59 ± 0.4	40 ± 0.3	60 ± 0.7	
		49±0.7	51 ± 0.4	42 ± 0.8	58 ± 0.6	0.02

## DISCUSSION

The current experimental study provided an overview about the role of FSCs in healing maternal renal damage during pregnancy where the GFP is set as the marker of fetal-origin cells in the maternal damaged part of kidney. The focus of the current study was on determining the role of FCSs in maternal renal function improvement.

It has been shown that the frequency of FMCT can be affected by histocompatibility between the mother and fetus (13). Approximately, 4 weeks post-gestation, FSCs can be detected in maternal circulation (14). In one study, the persistence of fetal leukocytes containing a Y body has been documented in the peripheral blood of women that were pregnant for the first time for more than 1 year after delivery (15). In another study in 1974, the persistence of fetal leukocytes in the maternal circulation was shown after gestation (16). In the study of Ciaranfi et al., notice-able male lymphocytes were detected in maternal blood of more than half of the women two years after delivery (17). In one study, it has been de-monstrated that although the frequency of FSCs was higher in murine lungs during pregnancy, the number of fetal cells decreased significantly 21 days after delivery (18). In a recent study, GFP-positive cells were detected in 20% of the maternal spinal cord in first pregnancy; while it reached to 80% after the third pregnancy percent (19). Pregnancy-associated progenitor cells which have properties similar to stem cells and are found in injured and/or diseased tissues, persist in maternal blood and organs for decades after delivery (3). Accordingly, the frequency of these progenitor cells reaches to its maximum level at 18-19th day of gestation (3).

FSCs have the capability to increase locally in the injured organ during and after pregnancy. In several maternal damaged tissues such as kidney, specific FMCT have been detected in both mice and humans. In the study of Khosro-tehrani et al. the liver of a pregnant murine model was injured by carbon tetrachloride and it was demonstrated that the number of FSCs increased in the affected organ (20). In one study on GFP mice, Streptozotocin (STZ) was injected in order to induce maternal beta cell injury and investigate the persistence of FSCs in the pancreas. The re-sults showed fetal DNA and EGFP+ cells in maternal bone marrow, kidney, pancreas and liver (21). Recently, it was concluded that cigarette smoke exposure in the pregnant mouse model, leads to FSC retention in the maternal injured lung (22). In another study, functional blood vessels has been formed in maternal inflammatory skin by fetal endothelial progenitor cells during pregnancy (23). In the study of Wang et al., it has been shown that fetal GFP-positive cells migrate to damaged liver and kidney that were exposed to alcohol and gentamicin, respectively (24).

It has been demonstrated that the morphological appearances of the FSCs within different mater-nal tissues was similar to differentiated maternal cells in mice during pregnancy (18).

Their results were in accordance with the outcomes of the current study in which we revealed that the morphological appearance of FSCs in the damaged kidney after three months of delivery was indistinguishable from the maternal counterparts. In spite of the fact that no specific tissue marker was applied in the present study which is considered as one of the its limitations, the improvement of renal function, and the successful cell culture with the renal cell protocol can approve the differentiation of these FSCs to renal cells.

In the study of Perin et al. stem cells from human amniotic fluids were injected into embryonic kidneys of murine. The results demonstrated the potential of these stem cells to differentiate into renal vessels culminating in early development of the kidney (25). In spite of the fact that it has been mentioned that FSCs have an ineffective role on maternal health, the results of the current study expressed opposite results as confirmed by DMSA scan and SPECT. It has been shown that no significant improvement was detected in renal function of control group in which unpregnant rats were evaluated. The role of FSCs in functional improvement was not evaluated in previous articles which is one of the benefits of the current study. SPECT study may be also helpful when the size of the renal damage in small which cannot be detected with planer study. In the present study, this method was applied for the first time to estimate the renal function of rats in the field of FMCT.

In the present study, we sought to determine the prolonged persistence of FSCs in damage part of maternal kidney even after the pregnancy. Therefore, we decided to apply GFP-positive trans-genic male rats to identify the FSCs more easily by their green color. Using this method, we achieved high sensitivity detection of FSCs in the section of the injured kidney. The ability of FSCs in migrating to damaged maternal tubular epithelial cells was demonstrated by immune histochemical analysis with anti-GFP antibody. The domiciliation of GFP-positive FSCs was confirmed by immunofluorescent microscopy according to their special immunofluorescent staining.

By PCR amplification of Y-chromosome sequences, we confirmed the persistence of FSCs in the blood circulation of the pregnant rat and the migration of Y-chromosome DNA of the male fetus to the damaged region of the kidney for further regeneration. In the present study, we confirm that FMCT may contribute to organ regeneration and repair. FSCs were only detected in injured region of the kidney and no FSC was distinguished in non-injured organs. These outcomes are in accordance with previous studies in which FSCs migrated to the maternal organs after induction of injury (20, 24). However, functional evaluation of the damaged organ was also studied to determine the role of FSCs in improvement of the impaired organ which was not previously evaluated in previous studies.

We developed a surgical model of renal injury during pregnancy in order to essay the fetal cell response in maternal damaged kidney. As the number of FSCs rise with the gestational age, we decided to perform the renal damage at GD 11 which is in the middle of gestational period. In the current study, we showed that FSCs improved renal function and increased the proliferative response in the damaged kidney compared to the normal one.

This study has also other limitations. Although the trend of improvement in functional parameters can ensure a persistent outcome in tissue repair, renal specific markers were not applied. However, renal function improvement, and detection of Y chromosome and GFP-positive cells in the sub-cultured medium indicated the ability of FSCs in migration from the peripheral circulation to injured maternal kidney and differentiate into renal cells. Considering the fact that serum creatinine and glomerular filtration rate are easier and cheaper modalities in bilateral renal injuries, their application was not feasible in this model in which unilateral renal damage was created. So, we focused on imaging techniques rather than these laboratory tests.

While medical research teams are now trying to estimate the advantages and disadvantages of FMCT in human societies, this study demonstrated a perfect result of this phenomenon in functional improvement of the maternal damaged kidney in rat model. Therefore, we concluded that fetal GFP-positive cells have the potential to persist after delivery and domiciliate in injured kidneys with dynamic respond. It can be also realized that FSCs in maternal tissues have the ability to act as a reservoir of stem cells and pregnancy is a protection against susceptibility to several diseases. However, further studies are required to estimate the role of FSCs in repairing the maternal damaged organs by introduction of FSCs to maternal organ after harvesting and expanding the cells in vitro. Additionally, further investigations are required to estimate the efficiency of FMCT in human pregnancies.

## CONCLUSIONS

In this study, we investigated the role of FSC migration and homing in their final destination in different target organs and tissue regeneration. We sought to characterize FSCs with multilineage potential that migrate to the maternal organs. The results of the current study revealed that FSCs play a crucial role in repairing maternal damaged kidney and improve its impaired function without prior in vitro manipulation. However, more studies are required to conclusively demonstrate the role of FMCT in human maternal damaged organs.

## Abbreviations

FSCs: = Fetal Stem Cells
GFP: = Green Fluorescent Protein
DMSA: = Technetium 99m-Dimercaptosuccinic Acid
SPECT: = Single-photon emission computed tomography
FMCT: = Fetal maternal cell trafficking

## References

[1] Baptiste N, Friedlander P, Chen X, Prives C (2002). The proline-rich domain of p53 is required for cooperation with antineoplastic agents to promote apoptosis of tumor cells. Oncogene.

[2] Dawe GS, Tan XW, Xiao ZC (2007). Cell migration from baby to mother. Cell Adh Migr.

[3] Pritchard S, Hoffman AM, Johnson KL, Bianchi DW (2011). Pregnancy-associated progenitor cells: an under-recognized potential source of stem cells in maternal lung. Placenta.

[4] Seppanen E, Fisk NM, Khosrotehrani K (2013). Pregnancy-acquired fetal progenitor cells. J Reprod Immunol.

[5] Bhattacharya N, Stubblefield P (2013). Fetomaternal Cell Trafficking: A Window into the Long-Term Health Effects of Treating Disease with Fetal Cell/Tissue Transplants?. Human Fetal Tissue Transplantation.

[6] Nassar D, Khosrotehrani K, Aractingi S (2013). Microchimerism in Mouse Pregnancy. The Guide to Investigation of Mouse Pregnancy.

[7] Khosrotehrani K, Johnson KL, Cha DH, Salomon RN, Bianchi DW (2004). Transfer of fetal cells with multilineage potential to maternal tissue. JAMA.

[8] Boudet J, Man NK, Pils P, Sausse A, Funck-Brentano JL (1978). Experimental chronic renal failure in the rat by electrocoagulation of the renal cortex. Kidney Int.

[9] Gibb IA, Hamilton DN (1985). An experimental model of chronic renal failure in mice. Clin Immunol Immunopathol.

[10] Gagnon RF, Ansari M (1990). Development and progression of uremic changes in the mouse with surgically induced renal failure. Nephron.

[11] Chow K-M, Liu Z-C, Chang TM-S (2003). Animal remnant kidney model of chronic renal failure revisited. Hong Kong Journal of Nephrology.

[12] Saiki RK, Gelfand DH, Stoffel S, Scharf SJ, Higuchi R, Horn GT (1988). Primer-directed enzymatic amplification of DNA with a thermostable DNA polymerase. Science.

[13] Nelson JL (2001). HLA relationships of pregnancy, microchimerism and autoimune disease. J Reprod Immunol.

[14] Thomas MR, Williamson R, Craft I, Yazdani N, Rodeck CH (1994). Y chromosome sequence DNA amplified from peripheral blood of women in early pregnancy. Lancet.

[15] Schröder J, Tiilikainen A, De la Chapelle A (1974). Fetal leukocytes in the maternal circulation after delivery. I. Cytological aspects. Transplantation.

[16] Tiilikainen A, Schröder J, De la Chapelle A (1974). Fetal leukocytes in the maternal circulation after delivery. II. Masking of HL-A antigens. Transplantation.

[17] Ciaranfi A, Curchod A, Odartchenko N (1977). Post-partum survival of fetal lymphocytes in the maternal blood. Schweiz Med Wochenschr.

[18] Khosrotehrani K, Johnson KL, Guégan S, Stroh H, Bianchi DW (2005). Natural history of fetal cell microchimerism during and following murine pregnancy. J Reprod Immunol.

[19] Zhang G, Zhao Y, Li XM, Kong J (2014). Fetal cell microchimerism in the maternal mouse spinal cord. Neurosci Bull.

[20] Khosrotehrani K, Reyes RR, Johnson KL, Freeman RB, Salomon RN, Peter I (2007). Fetal cells participate over time in the response to specific types of murine maternal hepatic injury. Hum Reprod.

[21] Sunami R, Komuro M, Yuminamochi T, Hoshi K, Hirata S (2010). Fetal cell microchimerism develops through the migration of fetus-derived cells to the maternal organs early after implantation. J Reprod Immunol.

[22] Vogelgesang A, Scapin C, Barone C, Tam E, Blumental Perry A, Dammann CE (2014). Cigarette smoke exposure during pregnancy alters fetomaternal cell trafficking leading to retention of microchimeric cells in the maternal lung. PLoS One.

[23] Nguyen Huu S, Oster M, Uzan S, Chareyre F, Aractingi S, Khosrotehrani K (2007). Maternal neoangiogenesis during pregnancy partly derives from fetal endotelial progenitor cells. Proc Natl Acad Sci U S A.

[24] Wang Y, Iwatani H, Ito T, Horimoto N, Yamato M, Matsui I (2004). Fetal cells in mother rats contribute to the remodeling of liver and kidney after injury. Biochem Biophys Res Commun.

[25] Perin L, Giuliani S, Jin D, Sedrakyan S, Carraro G, Habibian R (2007). Renal differentiation of amniotic fluid stem cells. Cell Prolif.

